# Mechanisms of ACL injuries in men’s football: A systematic video analysis over six seasons in the Qatari professional league

**DOI:** 10.5114/biolsport.2023.118024

**Published:** 2022-07-21

**Authors:** Raouf Nader Rekik, Roald Bahr, Flavio Cruz, Paul Read, Rod Whiteley, Pieter D’hooghe, Montassar Tabben, Karim Chamari

**Affiliations:** 1 Aspetar, Orthopaedic and Sports Medicine Hospital, FIFA Medical Centre of Excellence, Doha, Qatar; 2 High institute of sport and physical education of Sfax, University of Sfax, Tunisia; 3 Oslo Sports Trauma Research Center, Norwegian School of Sport Sciences, Oslo, Norway; 4 Institute of sport, exercise & health research, UK

**Keywords:** Soccer, Knee ligament, Risk factors, Etiology, Middle East, Prevention

## Abstract

To assess the mechanisms of ACL injury in male professional football players in Qatar across multiple seasons using systematic video analysis. 15 ACL injuries occurred in competition among the professional football teams that participated in an injury Surveillance Programme during 6 seasons (2013/2014 to 2018/2019). High-definition broadcast videos of these injuries were analyzed (49 views; 34 slow motion) by five analysts who independently described the injury mechanisms (situation, behavior, biomechanical characteristics) using validated observational tools. A knee valgus mechanism was observed in two-thirds of the cases (1 with direct contact to the knee, 3 with indirect contact (other body parts) and 6 with no contact). No visible valgus was reported in 2 of the direct knee contact injuries, while 3 cases of non-contact and indirect contact injuries were unclear. We observed 4 main categories of injury situation among those (n = 12) classified as non-contact/ indirect contact (multiple combinations were possible): pressing (n = 6), tackling or being tackled (n = 4), blocking (n = 3) and screening (n = 2). Direct contact injuries (n = 3) were suffered by 2 players during tackling and 1 whilst being tackled. Contact injuries represented only 20% of ACL injuries occurring during competition in Qatari professional soccer players. Independent of the playing situation, knee valgus was frequently observed (10/15 cases). Pressing was the most common situation (6/15 cases) leading to injury. Landing after heading was not reported in any of these ACL injuries.

## INTRODUCTION

Anterior cruciate ligament (ACL) injury for a professional football (soccer) player causes a significant burden [1–3]. On average, each professional football team will experience one ACL tear approximately every two seasons [2, 4]. A 15-year prospective study showed that 3 years after return to play following an ACL tear, only 2 out of 3 players still play at the highest level [5]. Furthermore, there is evidence to indicate that players with history of ACL injury are at a higher risk of sustaining secondary knee injuries [6, 7], early-onset knee osteoarthritis [8] and/or a shorter career [9, 10]. Effective ACL injury prevention programmes exist, but the incidence of injury remains high [5, 11, 12].

Understanding the mechanisms of these injuries could help clinicians refine measures aimed at preventing ACL injuries with longterm health, performance, and financial benefits [13–15]. Systematic video analyses are an important tool [16] which have been employed in several team sports over the past two decades, including handball, basketball and rugby [14, 17–22]. Two early studies analyzed ACL injuries including cases from football, but also from other sports [23, 24]. More recently, four studies have brought more insight into the football-specific mechanisms, although with some limitations related to study design, as low study power, selection bias, insufficient analytical methods including visual inspection of the videos [25–28]. Also, ACL injury incidence can vary according to the geographical region, and the time of year as climatic conditions vary [2, 29, 30]. No systematic video analyses have been performed in the Middle East where the climatic conditions are different (typically hotter and drier) compared to previous research. Fitness levels, training loads and championship intensity may vary as well.

The objective of this study was therefore to describe the mechanisms of ACL injury in professional Qatari football through systematic video analysis, over a 6-year period, based on published, systematic protocols [26], in an attempt to assess whether they reflect those observed in Europe.

## MATERIALS AND METHODS

### Injury inclusion and video recording

This study was conducted in accordance with previous research in European football players [26]. We identified cases based on the prospective Aspetar Injury & Illness Surveillance Programme [2, 31, 32] from the 2013/2014 through the 2018/2019 seasons. We extracted data on all complete ACL injuries occurring during official first team matches only. The clubs involved belonged to the first or second division of the football leagues competitions in Qatar and included 14, 16, 10, 15, 17 and 17 teams during the respective seasons. Data for the Aspetar Injury and Illness Surveillance Programme are recorded by medical staff assigned to each football club by Aspetar’s National Sports Medicine Programme [2, 31]. We identified 15 complete ACL tears; the clinical diagnosis was confirmed radiologically using MRI in all cases and high-definition (match) broadcast videos were available for all of them (49 views, 34 slow motion sequences). The number of camera views ranged from 1 to 6.

### Video processing

We used video-editing software (Adobe Premiere Pro, version 14.1.0, Build 116, Adobe Inc, San Jose, California, USA) to extract the relevant injury sequences from the entire match video into MP4 format. We created one single video for each injury, including a few seconds before and after the injury to assess the environmental and match conditions, injury situations and biomechanical variables. Each sequence included the video of the injury (normal speed) and all available replays with slow-motion (50% speed) from different camera angles (one single replay for 4 injuries, two for 4, three for 5, six for one). One additional injury was filmed with a wide shot with no replay, so we created a zoomed sequence with slow-motion (50% speed) using the same video editing software. Visualization of the videos was performed using QuickTime Player (version 7.7.4, Apple, Cupertino, California, USA) allowing easy frame-by-frame navigation. The different camera views were mounted successively through the single video sequence of each injury. The analysts would then choose the camera view(s) that allowed them to perform the best analysis.

### Video analysis

The video analyses were independently performed by five independent reviewers (two sport physicians, one orthopedic surgeon, one clinical research scientist and one physiotherapist) experienced in football, ACL injury, and biomechanics. The methodology used was based on previous systematic video analysis protocols [26]. We modified certain criteria, dividing the section “player action with the ball” and “duel type” into two additional options according to ball possession (Table 1). For each injury, the analysts determined which frame represented the initial contact (IC) of the foot of the injured leg with the ground. They then ticked check boxes related to categorical variables on injury information and circumstances, weather, pitch conditions, and biomechanical characteristics. The flexion angle of the hip, knee, and ankle was assessed at IC, IC+40 ms (one frame later) and IC+80 ms (two frames). We used the same definitions provided by a previous study on epidemiology of ACL injuries [2] to classify injuries as direct, indirect, or non-contact. The player’s speed was estimated as zero, low, high, or unsure according to the movement direction (horizontal, vertical). The analysts also assessed whether there was: hip abduction greater than 20°, knee valgus collapse, and/or an ankle eversion. Finally, two consensus in-person meetings were organized to review the five analysts’ forms for each injury. The videos were reviewed till agreement on all variables (except joint angles) was reached. A 3^rd^ in-person meeting was held by two analysts and one non-analyst co-author to check data completeness and accuracy.

**TABLE 1:** Categorical variables to describe the ACL injury circumstances and biomechanics.

Variable	Categories
Weather condition	Sunny, rainy, snowy, unsure

Pitch condition	Dry, wet, wet & muddy, unsure

Football-specific variables	

Playing situation preceding injury	Offensive, defensive, set play, other, unsure

Field location at injury	Defensive third, midfield zone 1, midfield zone 2, offensive third, unsure

Player action preceding injury (if no ball possession)	Heading, receiving, screening, turning, passing, shooting, blocking, clearing, other, unsure

Duel type (if no ball possession)	Heading, tackling other player, tackled by other player, collision (unintentional), blocking, screening, running, other, unsure, no duel

Player action preceding injury (if ball possession)	Being tackled, dribbling, heading, receiving, screening/shielding, turning, passing, shooting, blocking, clearing, other, unsure

If kicking	Kicking with right or left leg

Duel type (if ball possession)	Heading, tackling other player, collision (unintentional), blocking, screening/pressing, running, other, unsure, no duel

Player contact preceding injury	Yes, no, unsure

If contact, what type	Direct contact (to injured knee or injured leg), indirect contact (to uninjured leg, trunk, head/arm), other, unsure

Player contact at injury	Yes, no, unsure

If contact, what type	Direct contact (to injured knee or injured leg), indirect contact (to uninjured leg, trunk, head/arm), other, unsure

In balance at IC	Yes, no, unsure

If out of balance, what direction	Forward, backward, sideways

Biomechanical variables	

Player movement at IC	Forward, backward, sideways, other

Cutting angle at IC	Intended change of direction 0–30°, intended change of direction 30–90°, intended stopping or change of direction > 90°, unsure

Loading on one vs. two legs during the contact phase	One leg, two legs with equal load, two legs with main load on injured leg, two legs with main load on uninjured leg, unsure

Horizontal speed at IC	High, low, zero, unsure

Vertical speed at IC	High, low, zero, unsure

Trunk rotation	Toward injured leg, toward uninjured leg, neutral, unsure

Foot rotation at IC	Internal 0–45°, internal > 45°, external, neutral, unsure

Foot strike at IC	Heel, toe, flat, unsure

Knee flexion angle	

IC	Flexion/extension (+/-) straight knee = 0

IC + 40 ms	Flexion/extension (+/-) straight knee = 0

IC + 80 ms	Flexion/extension (+/-) straight knee = 0

IF	Flexion/extension (+/-) straight knee = 0

Hip flexion angle	

IC	Flexion/extension (+/-) straight hip = 0

IC + 40 ms	Flexion/extension (+/-) straight hip = 0

IC + 80 ms	Flexion/extension (+/-) straight hip = 0

IF	Flexion/extension (+/-) straight hip = 0

Ankle flexion angle	

IC	Plantar flexion/dorsal extension (+/-)

IC + 40 ms	Plantar flexion/dorsal extension (+/-)

IC + 80 ms	Plantar flexion/dorsal extension (+/-)

IF	Plantar flexion/dorsal extension (+/-)

Knee valgus collapse	Yes, no, unsure

Large hip abduction (> 20)	Yes, no, unsure

Ankle eversion	Yes, no, unsure

Any additional comments on the injury mechanisms in your own words	

### Statistical analysis

The data were analyzed using Microsoft Excel (version 16.0, Microsoft Office 365 ProPlus, Redmond, Washington, USA) and IBM SPSS statistics version 21.0 (SPSS Inc, Chicago, IL).

Descriptive analysis was performed for all categorical variables. The joint flexion angles were assessed for inter-rater reliability. To calculate the variability across all cases, we measured the intraclass correlation coefficient (ICC) estimates and their 95% confidence intervals for each variable/joint based on a mean-rating (k = 5), absolute-agreement, 2-way mixed-effects model. The ICC was of 0.715^c^ for the knee joint, 0.531^c^ for the hip joint and 0.651^c^ for the ankle joint at IC. We calculated for the 5 analysts’ measures of these joint angles: the mean, standard deviation, and standard error, along with the 95% confidence interval of the mean. The measurement correlation between analysts was poor, as the IC frame chosen by each analyst differed. Therefore, it was agreed to calculate the mean value of the 5 video frame numbers at IC selected by the 5 analysts (excluding the outliers). These frame images (as many frames as available video views for each injury) were shared again with the 5 analysts who reanalyzed them to recalculate the IC angles on the same images, which was intended to improve the measurement correlation between analysts. The joint flexion angles for IC were reported as the mean of individual estimates along with the standard error for the main injury situations (Table 2). Joint angle measurement at IC+40 ms, IC+80 ms and IF were not recalculated and then excluded from the study.

**TABLE 2:** Knee, hip, and ankle joint angles.

	Knee flexion (°)		Hip flexion (°)		Ankle flexion (°)		Injury situation

Case #	Mean flexion	SE	Mean flexion	SE	Mean flexion	SE	
**1**	58	9	48	6	3	5	Tackling other player

**2**	14	2	35	7	6	10	Tackled by other player Screening

**3**	14	4	41	6	6	6	Pressing

**4**	25	3	34	4	26	5	Blocking

**5**	29	5	36	4	16	6	Pressing Tackling other player Blocking

**6**	22	7	30	6	16	6	Pressing Running

**7**	21	2	25	3	5	2	Dribbling Running

**8**	26	2	40	4	3	3	Tackling other player

**9**	17	6	34	5	11	2	Pressing

**10**	26	9	16	1	9	3	Tackled by other player Screening

**11**	18	3	15	5	5	5	Not scored

**12**	18	3	36	5	10	8	Pressing Blocking

**13**	62	7	62	9	12	6	Pressing Pressing

**14**	17	10	39	5	7	7	Tackled by other player Blocking Collision

**15**	-	-	-	-	-	-	Tackling other player

Note: SE: Standard error. For injury 15, joint angles were not measured as the player was hidden by an opponent and a teammate in the analyzed frames.

The injury situation categories were not assessed for the one injury filmed with a wide shot. Joint angles were also not measured in another injury, as the player was masked by an opponent and a teammate in the three analyzed views.

### Ethical considerations

Injuries were recorded through the Aspetar Injury Surveillance Programme. Their use was approved by the Anti-Doping Laboratory Qatar Ethics Institutional review board (IRB E2017000252). No personal information regarding the participants was disclosed or used. The high-definition broadcast videos were obtained from Qatari television.

## RESULTS

Of the 15 injuries, 9 were to the right and 6 to the left knee; 13 occurred in the regular league and 2 in the Qatar Cups. The weather at the time of injury was usually sunny (except one case where the weather was unsure) and the pitch was always dry. Eight, four, and three injuries were classified as non-contact, indirect contact, and direct contact injuries, respectively. Eleven injuries occurred on defense, three on offensive, and one during a breakdown attack. In 12 of the 15 cases, the injured player was not in possession of the ball. The maximum loading at the time of injury was either solely on the injured leg (10/15) or on both legs (5/15) but with main loading on the injured leg.

### Injury situations

A knee valgus mechanism was observed in 10 cases (1 with direct contact to the knee, 3 with contact to other body parts and 6 with no body contact). No valgus was observed in 2 cases (both direct knee contact), while 3 were unclear. In all but two cases, the knee was slightly flexed (13 cases, 14° to 29°) at the time of injury (table 2). Of the 12 non-contact/indirect contact situations, we observed four main categories of injury situations (note: multiple combinations were possible): pressing (n = 6), tackling, or being tackled by another player (n = 4), blocking or stopping the ball from reaching another player (n = 3) and screening or protecting the ball with own body (n = 2). In one case, the player was injured while running and dribbling without interaction with any opponent. He did a cutting manoeuvre (30 to 90°) with the foot planted firmly on the ground involving rotational stress on the weight-bearing knee. In the 3 direct contact injuries, the player was tackling in 2 cases and being tackled in the other. No landing after heading situation was reported for any of the injuries.

### Injury situation for indirect and non-contact injuries (n = 12)

Of the 8 non-contact and 4 indirect contact injuries, there was one video with no contact, in which it was impossible to assess the situational pattern of the injury (one video incidence with no replay and no lateral view). Players were mainly running forward (11/12) and not jumping (10/12).

### Pressing (n = 6)

**FIG. 1:**
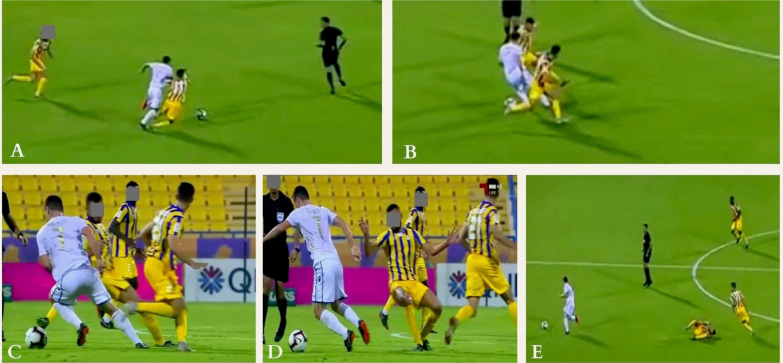
Indirect contact injury mechanism (Pressing) to the right knee: The injured player (in yellow) was running at high-speed trying to join a teammate in pressing an opponent (frame A). When he joined him, the opponent dribbled both by doing a sudden change of direction of 180° (frame B). The injured player tried to shoot the ball but missed it (frame C). He quickly stopped and changed his main loading from left to right leg while distracted by the ball causing a knee valgus and rotational motion (frame D). Then the player lost balance and fell down (frame E).

The injured players were most frequently in a defending situation, pressing another player, while not in possession of the ball (figure 1). Players were running forward, more often at a high speed (n = 4, low speed n = 2), with a variable cutting angle and torso rotation. They all displayed knee valgus. Both indirect contact cases were to the trunk/arm. Half of the players were off-balance sideways. The maximum loading during injury was on one leg in half the cases, with the other half on 2 legs but mainly loading the injured leg. There was a large hip abduction (> 20°) in half of the cases, with a moderate hip flexion (30 to 62°). The knee was slightly flexed (14 to 29°), except for one case. Ankle eversion was observed in only 2 cases. Foot external rotation was noted in 4 cases out of 6. The foot strike was mainly on the heel (n = 5).

### Tackling or being tackled (n = 4)

**FIG. 2:**
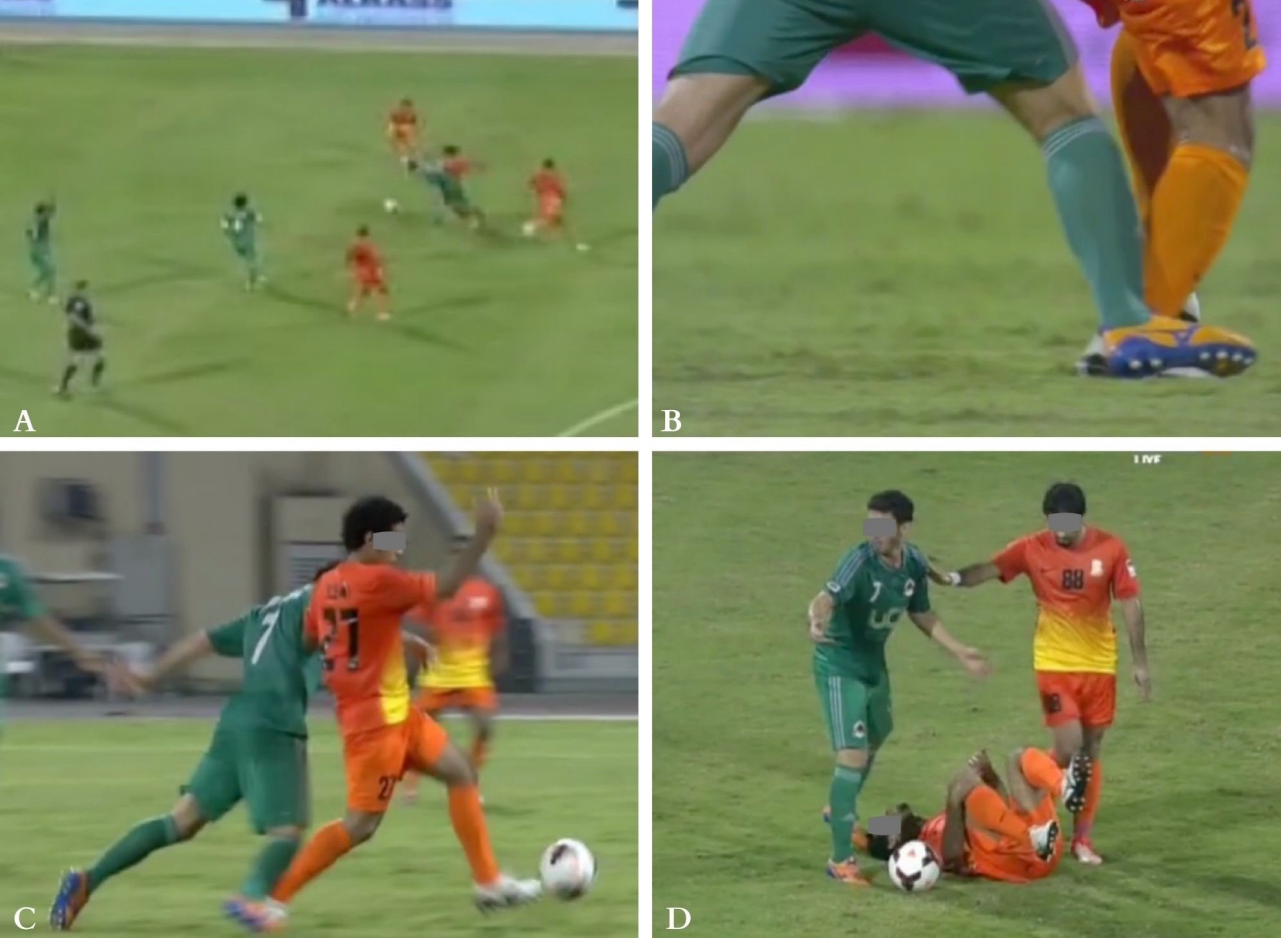
Indirect contact injury mechanism (tackled by other player) to the right knee: the injured player (in orange), gained possession of the ball from an opponent (frame A). The second player immediately tackled him and stepped on his foot (frame B). The injured player continued running forward with his right foot blocked behind on the ground (Frame C). He lost balance and fell (frame D).

The second more frequent injury situation was tackling from the lateral side (two cases in defensive situation) or being tackled (in a breakdown in an offensive situation), from behind in one case and laterally in the other (figure 2). Players were running forward with a variable cutting angle, in balance (except one case) and not jumping. The hip abduction was variable, while hip flexion was slight (16 to 36°). Knee flexion was slight (14 to 29°). The foot strike was variable but most frequently in external rotation (n = 3) and with no ankle eversion.

### Blocking (n = 3) and screening (n = 2)

**FIG. 3:**
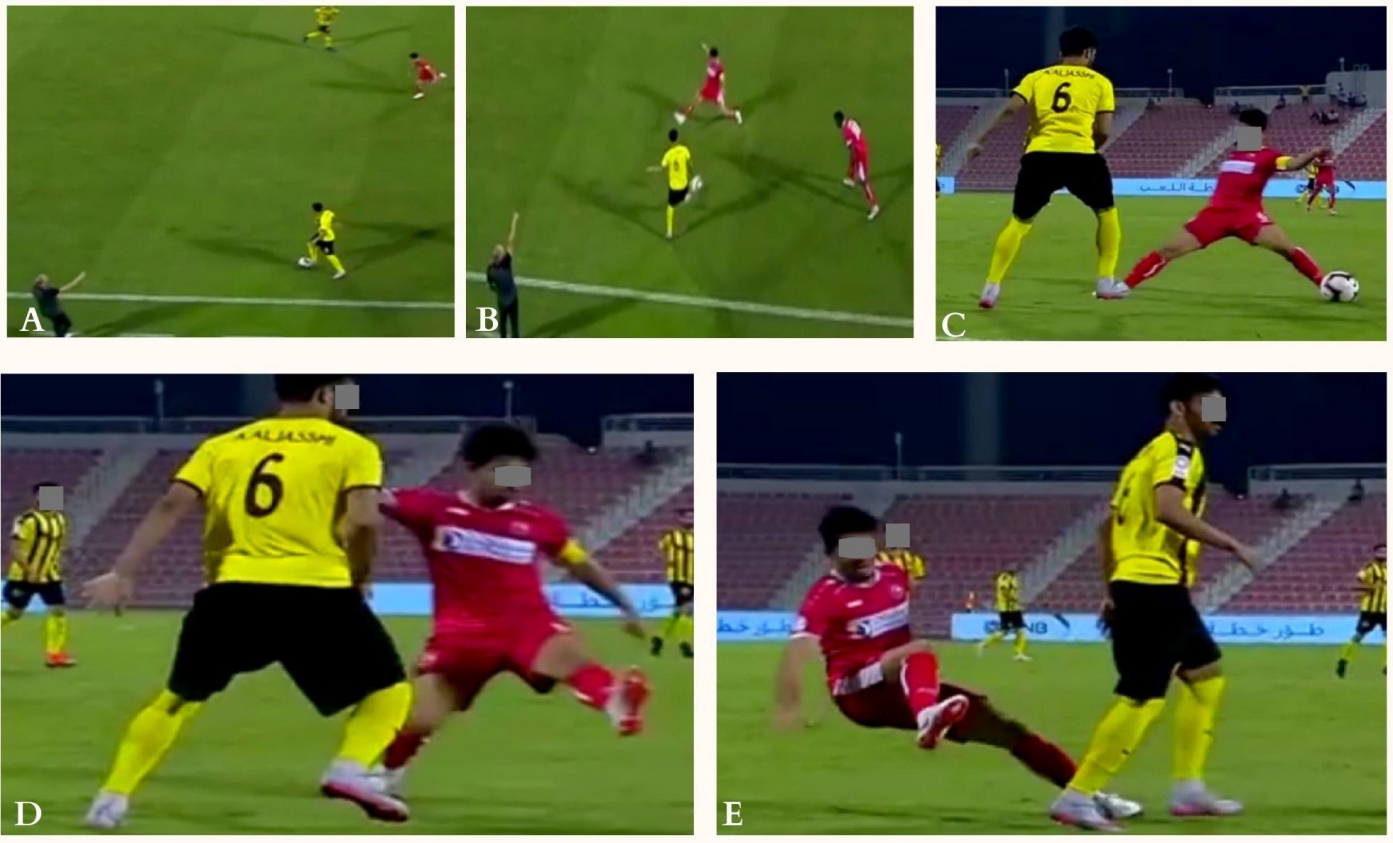
Indirect contact injury mechanism (blocking) to the right knee: The injured player (in red) was running at high-speed trying to stop the ball from being passed to another player (frame A). Then he kicked the ball while his right foot was planted on the ground with a large hip abduction (frame B) causing a knee valgus and rotational motion (frame C). Then the player lost balance and fell down (frame D).

**FIG. 4:**
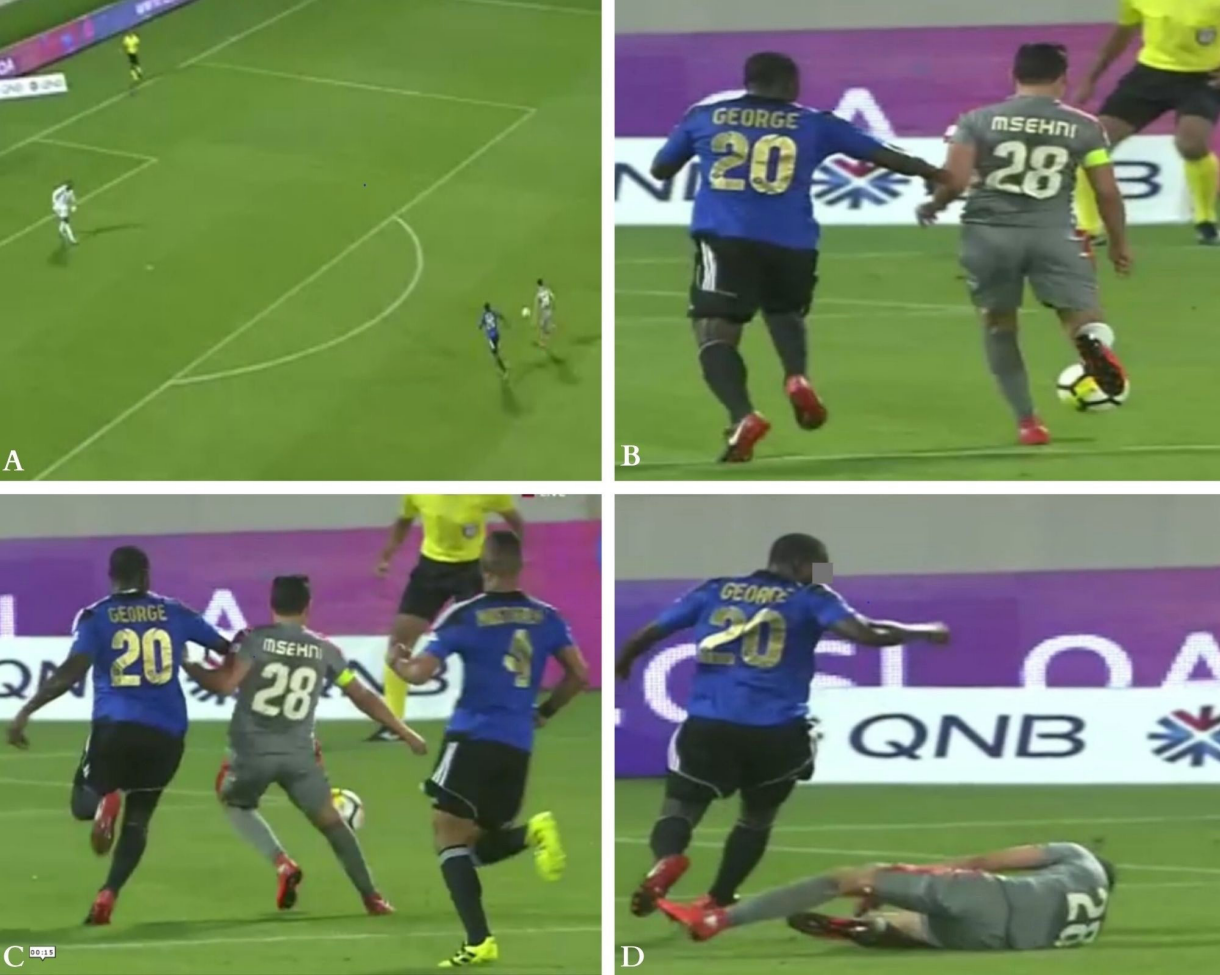
Indirect contact injury mechanism (Screening) to the right knee: The injured player (in grey), in offensive situation, was running forward at high speed, with ball towards the goalkeeper, alongside the defender (frame A). He was screening/shielding to protect the ball while running. During arm/trunk contact (frame B and C), when loading on his right foot (frame C), the injured player had a right knee valgus stress with compression mechanism causing the injury that resulted in his fall on the pitch and immediate call for medical help (frame D).

Blocking and screening were two other injury situations (Figure 3 and 4). The indirect contact was mainly to the trunk/arm in players moving forward (4/5). There was no clear trend for speed, cutting angle or losing balance. The torso rotation was neutral when screening and towards uninjured (n = 2) or injured leg (n = 1) when blocking. The hip abduction was variable and the hip flexion about 34 to 36° (except one case). The knee was slightly flexed (14 to 29°). The foot position was always in external rotation with a foot strike either on the heel or the toe.

### Injury situation for direct contact injuries (n = 3)

**FIG. 5:**
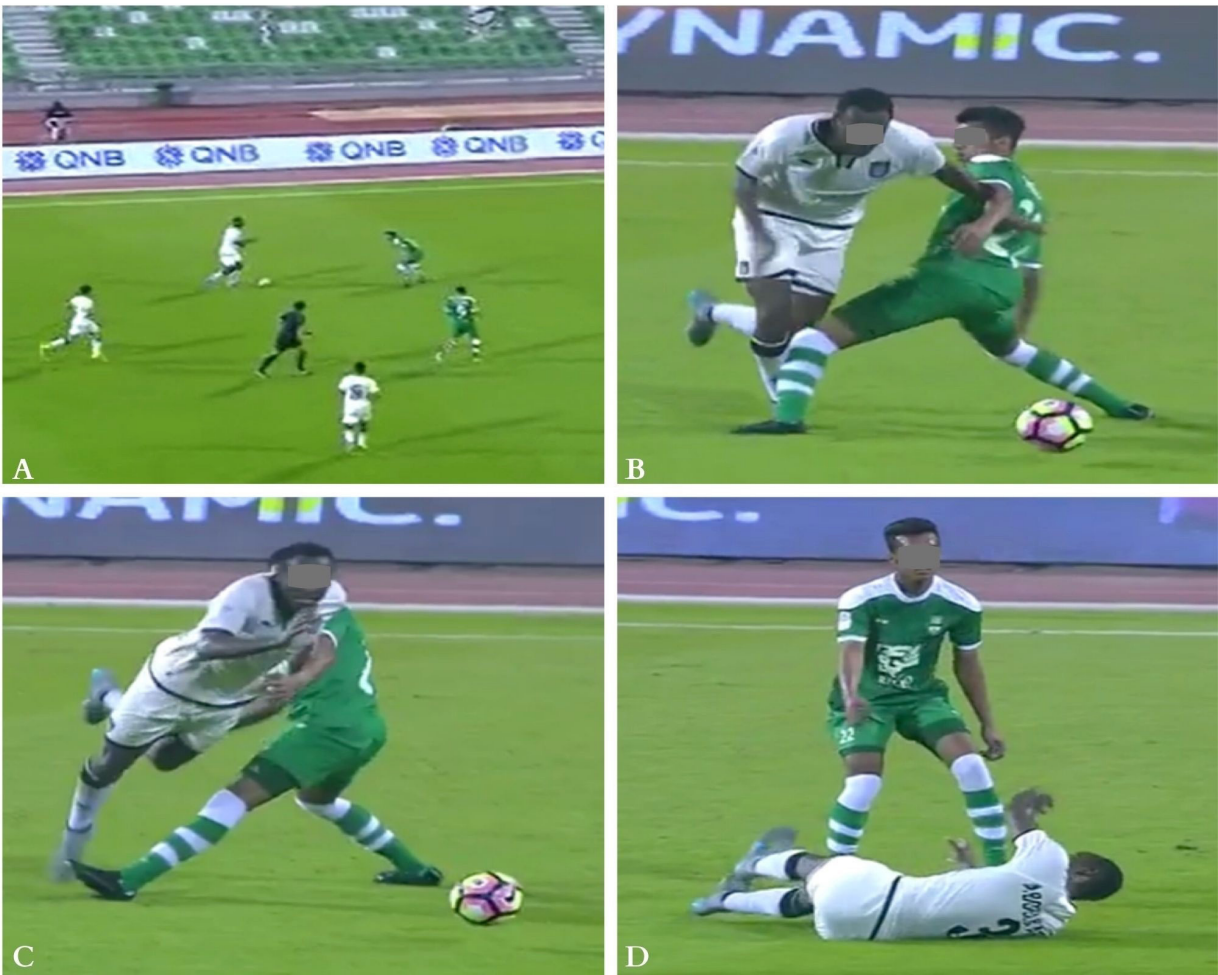
Direct contact injury mechanism (tackling other player) to the left knee: The injured player (in green), in defensive situation, making short forward and backward steps waiting for the striker to come in front of him (Frame A). Then the injured player stretched his left leg (in a weightbearing position) to tackle the opponent. Both players’ knees got in contact (frame B), causing a left knee compression mechanism in flexed position to the injured player. Following this contact, the injured knee went in extension (frame 3), the opponent fell down, the referee whistled a foul and the injured player continued to play (frame 4).

**FIG. 6:**
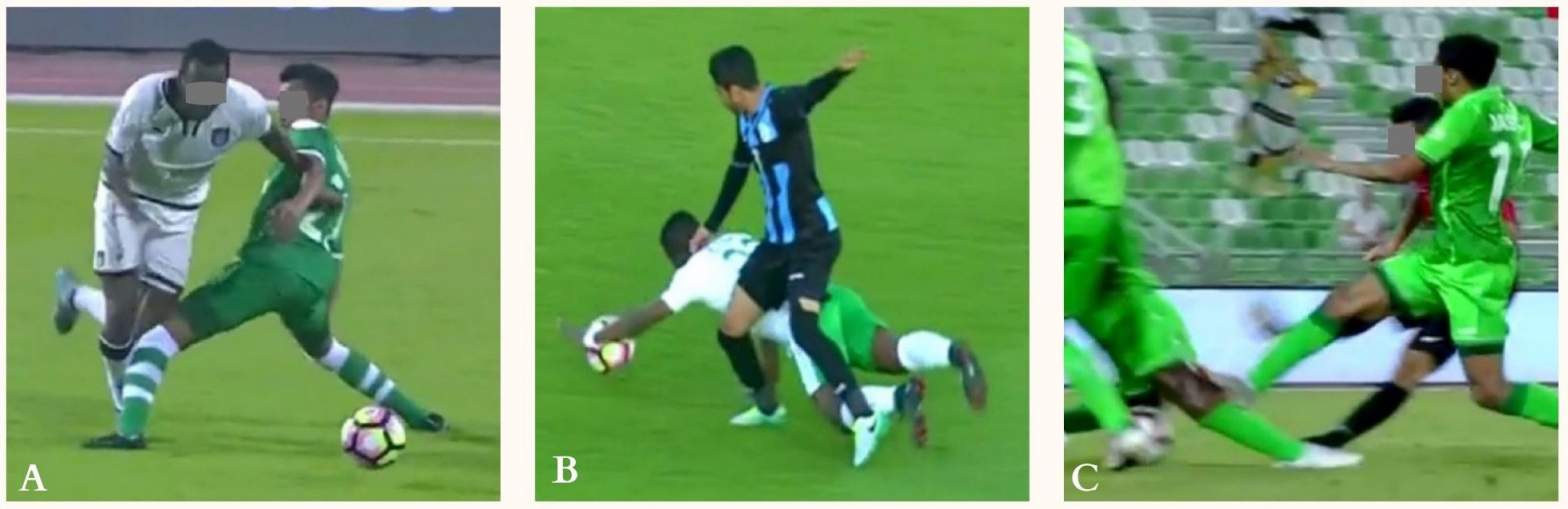
injury situations in direct contact injuries. Injured knee in frame A (Left knee, green Jersey player), frame B (Right knee, blue and black jersey player) and frame C (Right knee, green jersey player).

This direct contact to the injured knee involved players running either forward (n = 2) or a combination of backwards and sideways (n = 1), with low speed to defend against an opponent with the ball. There was no clear trend for the cutting angle or torso rotation. The 3 collisions involved tackling but included different situations (figure 5 and 6): 1. knee to knee contact involving anterolateral impact to the knee and leading to a knee compression mechanism in flexed position (58°); 2. body to knee contact with loss of balance, involving a postero-lateral impact to the knee and causing a knee valgus and an upper leg anterior translation; 3. foot to knee contact involving anterior impact and leading to knee hyper-extension. In the 3 cases, there was mainly a large hip abduction (2/3) with a flexed hip (39 to 48°). Foot rotation, foot strike and ankle eversion varied.

## DISCUSSION

This study described the ACL injury mechanism in professional Qatari football players over a 6-year period through systematic video analysis. A knee valgus mechanism was observed in two-thirds of the cases. Indirect and non-contact mechanisms were responsible for 12 out of 15 ACL injuries in which the 4 main injury situations were (with multiple combinations): (1) pressing, (2) tackling or being tackled, (3) blocking and/or (4) screening. All 15 injuries occurred in dry conditions, in accordance with previous research, suggesting that warm and dry conditions increase shoe-surface traction and thus are associated with higher incidences of non-contact ACL injuries compared to wet (soft) surfaces [29, 33, 34].

### Injury circumstances

In accordance with our findings, previous studies concluded that a non-contact mechanism is more frequent for ACL injuries in football [26, 35, 36]. Conversely, a recent study on professional Italian football players found a higher proportion of contact injuries (56%) [28]. The discrepancy across studies can be explained by a different definition of contact, whether directly to the knee or with other body parts (e.g. pushing an opponent or holding him by the shirt, defined as indirect in our study) [16], as well as different approaches from the collection of videos to sample analyses. Direct contact injuries, 20% in our study, were less frequently reported in the most recent papers [26, 28].

### Playing situations associated with ACL injury

In our study, 80% of ACL injuries occurred while defending; recent research reported more than two-thirds happening in this phase of play [25, 26, 28]. We observed four main injury situations, of which pressing was the most frequent (40%), consistent with the findings of others [26, 28]. The second mechanism was tackling or being tackled. Two new injury situations (blocking and screening) were observed. A one-legged jump landing has been reported as a common mechanism [26, 28], but was absent in our study. Contrary to a previous research on direct contact injuries [26], where all 3 tackling cases were tackled players with contact to the injured knee and suffering a valgus collapse, we observed a direct contact dynamic valgus in only one case out of three, in a tackling situation by the injured player. This could potentially be attributed to playing style variations [37] or anthropometric differences [28]. In our study, half of the indirect and non-contact injuries occurred in players losing balance, often while trying to shoot or stop the ball while being distracted by the ball or the opponent. Such situations are common with other sports like rugby [21] and American football [19] and should be practiced in controlled environment to prevent ACL tears. This can include perturbation, dual task or partial visual occlusion to increase the perceptual cognitive demands of the task. Furthermore, footwork and running technique training (neuromuscular and postural control) are key recommended elements in the prevention of this injury [38, 39], as well as addressing safe pressing and tackling technique.

### Lower limb biomechanics

Faulty mechanics during hazardous sporting movements, due to poor neuromuscular control, have been reported to cause a knee valgus stress and increased load on the ACL, increasing ACL injury risk [38, 40, 41]. These mechanics can result from lateral movement of the trunk, altered load distribution between limbs, and/or lack of movement control [38].

Altered motion in the frontal plane have been reported in literature [42]. In our study, the torso rotation showed no clear trend. Hip abduction (> 20°) was observed in 8 cases out of 15 (in addition to 2 unsure) while hip flexion varied. Similar to knee valgus-type loading, hip abduction motion from IC to the suspected moment of the ACL rupture has also been frequently reported [20, 26, 43, 44], with a planted laterally oriented foot [43, 45]. In our study, the foot was externally rotated in 60% of cases and ankle eversion occurred in only one case out of 3.

All indirect contact and 75% of non-contact injuries happened with the knee close to full extension at IC (< 29°). All, except 3 unsure cases, suffered a dynamic knee valgus stress identified by visual inspection. This is consistent with existing literature in football and other sports [14, 15, 26, 28, 46]. Previous research did not reach consensus whether knee abduction moments and greater angles are associated with greater risk of ACL injuries [47–50]. Moreover, vertical ground-reaction compression force increases during ground contact when the knee is less flexed and the trunk is in a more erect position. This has been shown to lead to an anterior tibial drawer and internal rotation, causing increased loads on the ACL [51]. In other studies, using the model-based image matching technique to assess knee joint motion, from IC to IC+40 ms, a 20° to 25° increase in mean knee joint flexion angle has been observed [20, 26, 28], reflecting sudden compressive forces at the knee joint. This finding was not assessed in our study as we considered only measurements at IC.

The above-described faulty mechanics are modifiable through inclusion of plyometrics, neuromuscular and strength training. A dynamic assessment could potentially recognize the alterations in the trunk and lower kinetic chain to address [38, 40]. Compliance of the football players is essential in these preventive programmes, as well as coach education to overcome obstacles (implementation in warm up) [52].

### Methodological considerations

Our study’s main strength is that it involves a homogeneous sample from our prospective injury and illness surveillance programme collected by club medical staff working for the same institution and following the same data collection protocol, aligned with the recent IOC consensus recommendations [53]. The diagnoses were also verified as ACL rupture was always confirmed on MRI and all cases went to surgery. All match injuries recorded during the observation period were captured on video, with high-definition quality in all cases but one. Furthermore, we followed the same approach and methodology as in previous research [14, 15, 26, 54]. The sample size, with fewer cases (15 injuries from official matches) than other studies, reflects the outcome from six consecutive seasons in a single country with a relatively limited number of teams and official matches when compared to European football leagues. Although unlikely to dramatically change the pattern from that observed, collecting a larger sample with data from several championships in the Middle East would provide a more granular picture. Thus, our data should be interpreted with some caution, but at least reflect the main mechanisms of ACL injuries in Qatar.

Furthermore, only official match injuries were reported, and these comprised a minority of the total injuries incurred in the league during this period (15 out of 51; training and friendly game injuries could not be included since no video footage was available). Future research should consider methods to describe training injuries, as well, since their mechanisms may differ from match injuries. Should this be the case, there will be important prevention implications as training circumstances are probably more amenable to change than matches. Additionally, these data only apply to adult males, so extrapolation to adolescents and females should be done with caution, if at all. Still, a recent study on female professional football players found similar injury situations and mechanisms as in males [55].

The number of camera views ranged from 1 to 6. In previous studies, the various camera views were manually synchronized based on different criteria and presented side by side on a single screen [26]. This could result in some misinterpretation. Thus, we implemented the different camera views successively through a single video sequence for each injury. The analysts would then choose the camera view(s) that allowed them to best perform the analysis. Only one injury included a single camera view. Frontal and/or sagittal views were not always available. One video, filmed only with a wide shot, was appraised for all variables but injury situation categories and a second video was not assessed for joint angles as the player at IC was hidden between a teammate and an opponent.

Moreover, we used 5 analysts with expertise in sport medicine coming from different backgrounds. We believe that this insured a better outcome of the organized consensus meetings.

Joint angles were estimated by visual inspection and could not been validated. Previous research has outlined the limitations of visual inspection compared to a three-dimensional motion analysis system [56], leading us to not include data for the joint angle estimates at IC +40 ms, IC +80 ms and IF. Joint flexion angles for IC were reported as the mean of individual estimates along with the standard error for the main injury situations. Consequently, the dynamic progression in time of joint angles and body motion between IC, IC+40 ms and IF was not estimated, limiting the findings to a static description at ground contact. Furthermore, using the images (as many as many camera views) interchangeably for each injury could cause a parallax error.

## CONCLUSIONS

This study provides insights about the injury mechanisms in Qatar, a country in the middle East, which is characterized by hot and dry conditions. A common feature of most injuries was that the knee was in valgus and close to full extension. Direct contact injuries were less frequent (20%) and caused by the injured player tackling or being tackled. The indirect or non-contact injuries were related to 4 main injury situations (with multiple combinations): (1) pressing, (2) tackling or being tackled, (3) blocking and/or (4) screening. In contrast to previous research, one-legged jump landing was not observed. Preventive measures should consider the findings of our study.
